# The Improper Disposal of Drugs and the Lack of Information About It Among a Highly Educated Population in Brazil: Analysis of the Factors Influencing Correct Disposal

**DOI:** 10.3390/pharmacy14020060

**Published:** 2026-04-15

**Authors:** Jamila Alessandra Perini, Thais da Silva Figueiredo, Mayara Calixto da Silva, Daniel Escorsim Machado, Jéssica Vilarinho Cardoso

**Affiliations:** 1 Research Laboratory of Pharmaceutical Sciences (LAPESF), Pharmacy Department (DepFarm), Rio de Janeiro State University (UERJ), Rio de Janeiro 23070-200, RJ, Brazil; thaisfigueiredo91@outlook.com (T.d.S.F.); jessica_vilarinho@yahoo.com.br (J.V.C.); 2 Postgraduation Program in Environmental Science and Technology, Rio de Janeiro State University (UERJ), Rio de Janeiro 23070-200, RJ, Brazil; 3 Postgraduation Program in Public Health and Environment, National School of Public Health (ENSP), Oswald Cruz Foundation (Fiocruz), Rio de Janeiro 21040-900, RJ, Brazil; 4 Clinical Analysis Research Laboratory (LAPAC), Pharmacy Department (DepFarm), Rio de Janeiro State University (UERJ), Rio de Janeiro 23070-200, RJ, Brazil; mayaracx_2010@hotmail.com (M.C.d.S.); danielescorsim@yahoo.com.br (D.E.M.)

**Keywords:** pharmaceuticals disposal, environmental health education, environmental hazard

## Abstract

The improper disposal of household pharmaceuticals is a global public health concern, posing risks to both human health and the environment and highlighting the need to raise public awareness. This study aimed to identify individual characteristics associated with the inappropriate disposal of household pharmaceuticals and to determine which individuals may require greater awareness. This cross-sectional study included 310 participants who completed an online questionnaire collecting sociodemographic and clinical information, as well as data regarding the participants’ use, storage, and disposal of medications. Most participants were female (74%), single (57%), had a university degree (81%), and were 34 ± 12 years old; 37% had some non-communicable disease (NCD), 85% used some medication, and 62% disposed of pharmaceuticals inappropriately. Having no undergraduate degree (OR = 4.4; 95% CI = 2.01–9.8), an absence of NCDs (OR = 2.5; 95% CI = 1.1–5.6), a lack of knowledge about reverse logistics (OR = 3.6; 95% CI = 1.7–7.6) or environmental risks (OR = 13.5; 95% CI = 1.5–125), and a lack of guidance from healthcare professionals (OR = 3.6; 95% CI = 1.2–10.6) were associated with inappropriate disposal. Although most respondents (88.6%) were aware of the negative environmental impacts of improper disposal, 69.7% did not know what reverse logistics was or where to find collection points (73.5%). These findings highlight the importance of environmental education for the effective implementation of reverse logistics for household pharmaceuticals.

## 1. Introduction

Despite the undeniable benefits of pharmaceutical products in disease treatment, they can also be harmful to human health and the environment, specifically when disposed of improperly [1,2]. Effective guidelines for the household disposal of unused or expired pharmaceuticals are still required [3], especially in developing countries [4,5], such as Brazil [6].

In Brazil, the improper disposal of active pharmaceutical ingredients and endocrine-disrupting compounds that can act upon water and soil poses a significant challenge and has been a subject of evaluation since the 1990s [7,8,9,10,11]. In 2010, the National Solid Waste Policy (PNRS) was created, and a reverse logistics system was implemented [12]. To raise public awareness, the Resolution of the Collegiate Council (RDC) 222 of 2018 specified how to correctly dispose of pharmaceutical products, utensils, and excreta from patients undergoing drug treatment [13]. It was not until 2020 that reverse logistics was introduced in Brazil, determining that expired or unused household pharmaceuticals, as well as their packaging, should be directed to specialized collection points and then returned to the respective sector for environmentally safe disposal [14]. However, this process has not yet been fully disseminated and implemented in Brazil; it requires the creation of public policies that can guarantee the dissemination of this practice.

The household storage of pharmaceuticals itself is a global public health issue. The increased prevalence of clinical conditions (such as mental health disorders and acute and chronic diseases) and the impact of the pandemic have led to higher medication consumption and, consequently, higher medication storage at home [15]. Purchasing pharmaceutical products in excessive quantities results in surplus medications that may be stored or disposed of improperly and contributes to inappropriate self-medication, accidental intoxication, or non-adherence to the correct drug therapy [1]. In addition, storing medications at home can cause contamination or altered quality and effectiveness due to improper storage in kitchens or bathrooms, which exposes them to heat and moisture [1,16]. Therefore, it is crucial to implement programs by which to safely dispose of unused or expired pharmaceuticals kept in households and to generate data on their consumption, storage, and disposal, with the aim of reducing their negative health and environmental effects.

In view of this, this study aimed to describe the main demographic characteristics of a sample of Brazilian individuals recruited during the second year of the COVID-19 pandemic and their knowledge and practices regarding the disposal of household medications. In the future, we intend to propose health education initiatives on the correct disposal of household medications.

## 2. Materials and Methods

### 2.1. Study Design and Population

This work was approved by the Human Research Ethics Committee of the National Institute of Traumatology and Orthopedics—INTO (protocol number 4,522,437, February 2021). A cross-sectional observational survey was conducted using snowball and non-probabilistic sampling between May 2021 and July 2022, disseminated through the official pages of the Pharmaceutical Sciences Research Laboratory—LAPESF, State University of Rio de Janeiro (UERJ) (https://lapesfuerjzo.my.canva.site/ and @lapesf.jamila.perini on Instagram, accessed on 8 January 2026) and through the personal social networks of the research team.

Participants signed a free and informed consent form and completed the questionnaire through the Google Forms platform. The inclusion criteria were participants 18 years of age or older who reside in Brazil. This was verified through mandatory self-reported questions about date of birth and country of residence at the beginning of the survey. In the event of duplicates being identified by the full name, phone number, and email addresses of the participants, only the most recent response was considered.

### 2.2. Questionnaire

The questionnaire was created online using the Google Forms platform (https://docs.google.com/forms/d/e/1FAIpQLSfMnZRzKKu_SU4FVRxVRVbvZDQTW9uQVkJAg4zrI8kjkHS9OQ/viewform?usp=dialog, accessed on 6 April 2026) and was structured as follows: (1) an introductory section covering sociodemographic characteristics such as gender, age, marital status, and educational level; (2) a clinical section to determine if individuals had NCDs such as cardiovascular, respiratory, and autoimmune diseases and/or COVID-19; (3) a pharmaceuticals section to determine if individuals used medications or vitamins and if they were stored at home; and (4) a section regarding knowledge of reverse logistics and potential risks of inappropriate disposal of pharmaceutical products.

Those who reported disposing of pharmaceuticals at collection points were classified as “Appropriate disposal”, while those who reported disposing of them in household waste or sewage were considered “Inappropriate disposal”.

### 2.3. Statistical Analysis

Continuous variables were expressed as mean ± standard deviation (SD). Categorical variables were expressed as number (n) and percentage (%), and differences between proportions were assessed using Pearson’s chi-squared test (χ^2^) or Fisher’s exact test when appropriate. Variables with statistical significance in the simple logistic regression model (*p* > 0.20) were included in the multiple logistic regression, with *p* < 0.05 remaining significant. The magnitude of the association between the variables and inappropriate pharmaceuticals disposal was assessed using the odds ratio (OR) with a 95% confidence interval (CI). The goodness of fit of the final model was assessed using the Hosmer–Lemeshow test. Missing data were excluded from the analysis. All analyses were performed using IBM SPSS 20.0 Statistics for Windows (SPSS Inc., Chicago, IL, USA), with a statistical significance level of 0.05.

## 3. Results

**Figure 1:**
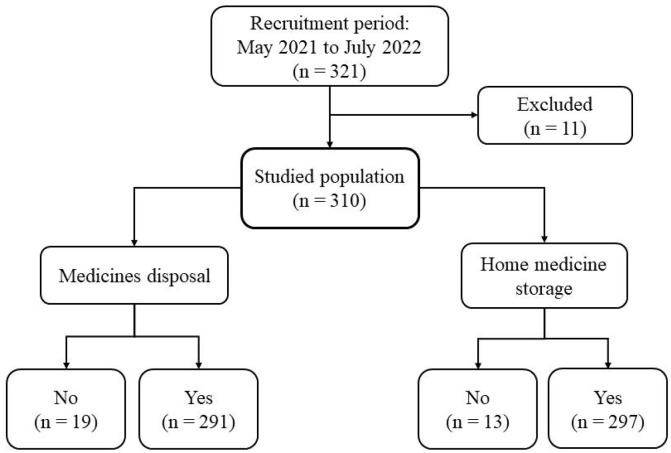
Flowchart of the population studied and their medication storage and disposal habits (n = 310).

A total of 321 responses to the online questionnaire were received, of which 11 were excluded due to duplication. This resulted in data from 310 individuals being included in the study. The majority (95.8%) stored some pharmaceutical products at home, and only 6.1% did not dispose of household pharmaceuticals as they tended to store them even after they had expired (Figure 1).

Most individuals were female, single, with an undergraduate or postgraduate degree, and had a mean age of 34 ± 12 years (median = 30 and range = 18–84 years), with approximately 73% aged 18–39, differing from the Brazilian Institute of Geography and Statistics (IBGE) statistics (2022 Census) with respect to sex, age, and education, while the proportion of single individuals was similar. Additionally, while approximately 63% of participants had no NCDs, 85.2% were taking medication. The most common types of medication were vitamins, painkillers, contraceptives, and antibiotics (Table 1).

Regarding pharmaceuticals storage and disposal habits, around 50% of respondents reported storing medications in locations such as kitchens and bathrooms, and 62% reported inappropriate disposal practices. Almost 90% of participants were aware of the risks to the environment caused by improper disposal of pharmaceuticals. However, only around 30% of the population said they were aware of reverse logistics for these products. In addition, nearly 80% did not receive adequate information from a doctor or healthcare professional on how to properly dispose of unused or expired home pharmaceuticals (Table 2).

All sociodemographic and clinical characteristics, medication use habits, storage practices, and knowledge of reverse logistics were compared between individuals according to their pharmaceutical disposal habits, i.e., those who used reverse logistics (n = 110) and those who disposed of them in domestic waste or sewage (n = 181). Therefore, to evaluate the magnitude of the association between the characteristics of the study population and their disposal practices, two logistic regression models were proposed, including the variables with *p*-values < 0.20. The simple logistic regression revealed that individuals with an undergraduate degree in a health profession, at least one NCD, knowledge of reverse logistics and environmental risks, or those who used any medication or received guidance from a healthcare professional were associated with the appropriate disposal of pharmaceutical products (Table 3).

After adjusting for confounding variables using multiple logistic regression, only the habit of using and storing medicines at home was not associated with disposing of medication at collection points. All the other characteristics of the population studied were associated with medication disposal practices (Table 3). Thus, the variables associated with the disposal of expired or unused household medicines in the sewer system and/or household waste were: a lack of an undergraduate degree in a health-related field (OR = 4.4; 95% CI = 2.01–9.8), the absence of NCD (OR = 2.5; 95% CI = 1.1–5.6), a lack of knowledge of reverse logistics (OR = 3.6; 95% CI = 1.7–7.6) or environmental risks (OR = 13.5; 95% CI = 1.5–124.9), and the absence of guidance from a healthcare professional (OR = 3.6; 95% CI = 1.2–10.6). In addition, a sensitivity analysis excluding variables with more than 10% missing data showed that the associations remained statistically significant in a similar direction and magnitude. The Hosmer–Lemeshow test for the adjusted model was not statistically significant (*p*-value = 0.54), indicating that there was no statistical evidence of loss of goodness of fit.

## 4. Discussion

During the COVID-19 pandemic, there was a significant increase in the consumption and storage of pharmaceuticals in households due to the uncertainty of the period, the ease of acquisition, social distancing, lockdowns, and willingness to self-medicate. Therefore, the nation faced different, specific, and novel problems, which also contributed to the high number of people who store pharmaceuticals at home [15,17]. In the present study, almost 96% of people stored them at home and this scenario. This, combined with the improper disposal of these products, is a growing public health problem that harms human, animal, and environmental health around the world [1,2,12]. Thus, this study, involving 310 volunteers, described the variables associated with the disposal of home pharmaceuticals during the second year of the COVID-19 pandemic. Most of those surveyed were between 18 and 39 years old, female, unmarried, and highly educated. Despite the limitations of the study’s sampling methodology, the 2022 IBGE Census indicates that the current Brazilian population also comprises mainly women in the 18–39 age group. However, only a small percentage of the current Brazilian population has a high level of education, which reinforces the selection bias of the study. Nevertheless, this highlights the importance of this study and is a further cause for concern, as even highly educated individuals have been shown to be unaware of the correct way to dispose of household drugs.

Although most participants had no NCDs, almost all individuals had pharmaceutical products at home and 62% disposed of them improperly. In addition, approximately 70–80% of participants perceived a lack of information about the safe disposal of pharmaceutical products and a lack of guidance from healthcare professionals. Disposing of pharmaceuticals at a collection point was associated with individuals who had a bachelor’s degree in a health profession, at least one NCD, knowledge of reverse logistics and environmental risks, or instructions from a healthcare professional. Therefore, we suggest that public education efforts on proper medication disposal should particularly target individuals without NCDs and those not working in the healthcare sector. Despite these associations, it is important to note that some confidence intervals were relatively wide, indicating limited estimate precision, likely due to missing data and small subgroup sizes; therefore, these findings should be interpreted with caution.

During the pandemic, many turned to the internet due to various restrictions [18,19,20]. Our questionnaire was created using Google Forms, making it easily accessible via mobile phone. The questionnaire was disseminated by pharmacists and teachers, and most participants were therefore either undergoing or had completed higher education. This can be explained by the non-probabilistic sampling method applied, which cannot guarantee the representativeness of the study sample. Importantly, as the present study’s sample predominantly comprises individuals with higher educational attainment and, consequently, greater health awareness, the findings may reflect a conservative bias, potentially underestimating the true scale of inappropriate pharmaceutical product disposal in the general population. It is likely that the prevalence of improper storage and disposal practices is higher in the broader population, meaning that the extent of the problem in Brazil may be underestimated by this study. Additionally, the recruitment strategy employed in this study may have resulted in a more homogeneous and specific sociodemographic sample, thereby increasing the risk of selection bias. Despite these limitations, the snowball and non-probabilistic sampling methods were considered safer and more applicable due to the circumstances in which the present work was carried out, caused by the ongoing global pandemic. As with any study, we recognize that there are limitations, primarily in the methodology used to select research volunteers. This involved a snowball sampling approach (whereby each volunteer distributes questionnaires to their friends and followers) and non-probabilistic sampling (whereby self-recruited participants respond to an online questionnaire distributed via the personal social networks of the research team, primarily on Instagram and email). For instance, the risk of recall bias cannot be ignored, especially since the questionnaire did not provide explanations for any of the questions.

Regarding disposal and knowledge of reverse logistics, although most participants (~89%) were aware of the environmental impact of improper pharmaceutical disposal and were healthcare professionals, 70% did not know what reverse logistics was and/or where collection points were located. A study conducted during the COVID-19 pandemic period with 187 drug users found that 65% were healthcare professionals and approximately 78% were aware of the environmental consequences of improper disposal, confirming our findings. A large proportion of the population (~62%) still dispose of medications inappropriately [6]. Corroborating our findings, another study with 343 undergraduate students showed that the prevalence of proper disposal was higher among healthcare students (25–64%) compared to engineering students (0%) [16]. This result was expected, as healthcare professionals need guidance during their studies, preparing them for encounters with pharmaceuticals throughout their careers.

The presence of NCDs was associated with the correct disposal of home pharmaceuticals. It is therefore plausible that these individuals received some guidance from healthcare professionals on the most appropriate disposal method [3]. To the best of our knowledge, there are no analytical studies that have examined the relationship between the presence of NCDs and pharmaceutical disposal practices. Most studies regarding home storage and disposal are descriptive, with few performing statistical association analyses, such as those performed in this study. A recent review study evaluated factors associated with the disposal of home pharmaceuticals in surveys conducted worldwide [3]. Of the 20 selected surveys conducted, only 4 performed statistical analysis to investigate factors associated with disposal. Among studies adopting an analytical approach in different populations, some findings are consistent with those of the present study, such as those pertaining to the lack of information on the proper disposal of home pharmaceuticals [4,21].

Inappropriate disposal of pharmaceutical products can contaminate water and soil, affecting aquatic life, flora, and fauna. It can also contribute to antimicrobial resistance in microorganisms and have a negative impact on biodiversity, representing a major threat to public health [22]. Drugs in the environment, such as antibiotics and contraceptives (estrogens), can produce toxic by-products that break down slowly, causing teratogenic, mutagenic, and carcinogenic effects. Furthermore, if waste is burned improperly in rural areas, it can release toxic gases, posing additional risks to human health and the environment [23]. Therefore, it is crucial to implement adequate reverse logistics measures for pharmaceutical products to protect soil, water, biodiversity, and consequently, human health.

In developing countries such as Brazil, studies on the reverse logistics, storage, and disposal of pharmaceuticals are even rarer [3,24], but this topic has been increasingly recognized [2,25,26]. In 2020, during the pandemic period, a decree on the reverse logistics of home pharmaceuticals was created [27,28], although the main global concern was exclusively the adoption of preventive measures for, as well as the diagnosis and treatment of, COVID-19 [29,30]. Nevertheless, it is estimated that only 2% of Brazilian pharmacies have collection points for expired or unused products [31], highlighting the main problems for the effectiveness of the reverse logistics system created in 2020. Therefore, it is essential to strategically establish visible and accessible collection points in pharmacies, hospitals, health centers, public institutions, and even schools or universities, in accordance with Brazilian legislation (e.g., Decree No. 10,388/2020), and to develop strategies to disseminate knowledge about the reverse logistics of home pharmaceuticals to the entire population. For example, the US Food and Drug Administration (FDA) produced a video about how to safely dispose of unused or expired medicines, presented the available drug disposal options, and provided instructions on how to properly dispose of these medicines [32]. Additionally, healthcare professionals and home medication vendors should undergo training and be required to provide their patients and customers with information about reverse logistics.

Educational tools, combined with public campaigns emphasizing environmental and health risks, can make technical and complex information more accessible and engaging for the general population, help combat misinformation, and promote community knowledge and participation in decisions regarding the proper storage and disposal of pharmaceutical products. Preparing the public in this way is crucial to ensure proper pharmaceutical disposal, particularly in the event of future pandemics. To achieve this, we recommend targeted digital campaigns through social media and health apps, particularly for high-risk groups as defined by age, educational level, and health status. Additionally, counseling on proper pharmaceutical product disposal during medical consultations and pharmacy visits can help address the reported 80% gap in professional guidance.

The world must be prepared for, and aware of, the proper storage and disposal of pharmaceuticals, especially in the event of a new pandemic, to ensure that people continue to address these important issues that can affect human health. Therefore, the dissemination of scientific information on the subject by researchers, teachers, and the government is essential.

## 5. Conclusions

Individuals in Brazil without a degree or an NCD who were unaware of reverse logistics or environmental risks and who did not receive instructions from healthcare professionals were more likely to dispose of pharmaceuticals inappropriately. In addition, despite working in the healthcare sector, most respondents in this study were unaware of reverse logistics for pharmaceuticals and the collection points for expired or discarded products. These findings highlight the critical need for effective environmental education on the proper disposal of pharmaceuticals, particularly in Brazil and other developing countries.

## Figures and Tables

**Table 1:** Sociodemographic and clinical features of the study population (n = 310).

Characteristics ^1^	n	%	Characteristics	n	%
Sex			NCDs ^3^		
Female	229	73.9	Hypertension	59	19.0
Male	81	26.1	Respiratory diseases	26	8.4
Age (years)			Obesity	22	7.1
18–29	143	48.3	Hypo/Hyperthyroidism	19	6.1
30–39	72	24.3	Diabetes mellitus	12	3.9
40–49	39	13.2	Autoimmune diseases	8	2.6
50–59	29	9.8	Others	22	6.5
≥60	13	4.4	None	196	63.2
Missing	14	4.5	Medications in use ^4^		
Marital status			Vitamins	181	58.4
Single	176	56.8	Painkillers	121	39.0
Married	113	36.5	Contraceptives	102	32.9
Divorced	15	4.8	Antibiotics	66	21.3
Widower	6	1.9	Antidepressants	49	15.8
Educational level ^2^			Antihypertensives	44	14.2
Elementary	5	1.6	Antivirals	24	7.7
High school	53	17.1	Hormones	14	4.5
Undergraduate	166	53.6	Sedatives	12	3.9
Postgraduate	86	27.7	Others	15	4.7
			None	46	14.8

^1^ According to the 2022 Census by the Brazilian Institute of Geography and Statistics (IBGE), the current Brazilian population of 203,080,756 comprises 51.5% women. The age distribution is as follows: 38% aged 18–39 years; 14% aged 40–49 years; 12% aged 50–59 years; and 16% aged 60 years or over; 18.4% have a higher education qualification; approximately 49% are single, 39% are married, and 12% are divorced or widowed. ^2^ Categories include complete and incomplete education. ^3^ Participants can have more than one non-communicable disease (NCD). ^4^ Participants can use more than one medication.

**Table 2:** The knowledge and habits of the studied population with regard to the disposal of pharmaceuticals (n = 310).

Characteristics	n	%	Missing Information n (%)
Pharmaceuticals storage ^1^			
Bedroom/living room	178	57.4	0 (0)
Kitchen	128	41.3	
Bathroom	30	9.7	
Does not store	13	4.2	
Pharmaceuticals disposal			
Household waste	161	51.9	0 (0)
Sewage	20	6.4	
Collection point	110	35.6	
Do not dispose	19	6.1	
Knowledge of reverse logistics ^2^			
No	209	69.7	10 (3.2)
Yes	91	30.3	
Knowledge of environmental risks ^3^			
No	29	11.4	56 (18.1)
Yes	225	88.6	
Professional guidance ^4^			
No	219	79.6	35 (10.3)
Yes	56	20.4	

^1^ Participants can store medications in more than one place. ^2^ Question: “Have you ever heard of the reverse logistics system for medications?” ^3^ Question: “Did you know that the environment can be harmed by residue from improper disposal of medications?” ^4^ Question: “Have your doctor or healthcare professional given you any information about the proper way to dispose of unused or expired medicines?”

**Table 3:** Analysis of associations between medication disposal practices and the characteristics of the study population (n = 291).

Characteristics	Appropriate Disposal ^2^ n (%)		*p*-Value ^3^	Crude OR (95% CI)	*p*-Value ^4^	Adjusted OR (95% CI) ^5^	Missing Information n (%)
	Yes (n = 110)	No (n = 181)					
Health undergraduate degree ^1^							
No	22 (23.9)	97 (64.2)		Ref		Ref	0 (0)
Yes	70 (76.1)	54 (35.8)	<0.001	0.21 (0.09–0.31)	<0.001	0.23 (0.10–0.50)	
NCDs							
No	61 (55.5)	123 (68.0)		Ref		Ref	0 (0)
Yes	49 (44.5)	58 (32.0)	0.03	0.59 (0.36–0.96)	0.02	0.40 (0.18–0.88)	
Medication use							
No	9 (8.2)	33 (18.2)		Ref		Ref	0 (0)
Yes	101 (91.8)	148 (81.8)	0.02	0.40 (0.18–0.87)	0.25	0.48 (0.14–1.68)	
Home pharmaceuticals storage							
No	11 (10.1)	7 (3.9)		Ref		Ref	1 (0.3)
Yes	98 (89.9)	174 (96.1)	0.03	2.79 (1.05–7.43)	0.31	2.33 (0.46–11.8)	
Knowledge of reverse logistics							
No	45 (41.7)	147 (85.0)		Ref		Ref	10 (3.6)
Yes	63 (58.3)	26 (15.0)	<0.001	0.13 (0.07–0.22)	0.001	0.28 (0.13–0.60)	
Knowledge of risks to the environment from improper disposal							
No	1 (0.9)	28 (22.2)		Ref		Ref	55 (23.3)
Yes	109 (99.1)	98 (77.8)	<0.001	0.03 (0.004–0.24)	0.02	0.07 (0.01–0.69)	
Professional guidance							
No	68 (68.7)	142 (88.8)		Ref		Ref	32 (12.4)
Yes	31 (31.3)	18 (11.2)	<0.001	0.28 (0.14–0.53)	0.02	0.28 (0.09–0.82)	

NCDs: non-communicable disease. OR: odds ratio. CI: confidence interval. ^1^ n = 243; 48 individuals only completed high school or elementary school. ^2^ Question: “Where do you dispose of expired and/or unused pharmaceuticals in your home?” Answer choices: household waste; sewage; collection point; or do not dispose. ^3^ *p*-value = Pearson’s chi-square test (χ^2^). ^4^ *p*-value = Pearson’s chi-square test (χ^2^) from adjusted logistic regression model. ^5^ Adjusted for variables with statistical significance in the simple logistic regression model (health undergraduate degree, NCDs, medication use, home pharmaceuticals storage, knowledge of reverse logistics, knowledge of risks to the environment from improper disposal and professional guidance).

## Data Availability

The data presented in this study are available on request from the corresponding author.
